# Progress in Surgery

**Published:** 1896-02-29

**Authors:** 


### Progress in Surgery.

### HERNIA.

**The Coverings of the Sac.**—The long-standing belief that a hernial sac in descending the inguinal canal acquires a covering from the sheath of transversalis fascia (infundibuliform fascia), which envelopes the spermatic chord, has been recently assailed by Dr. John Packard of Philadelphia.[1] More than twenty dissections have been made by this observer to discover whether the disposition of the fascia about the chord at the inner ring favours the descent of a hernia along the course of the chord. Dr. Packard found "no such arrangement as would seem to predispose to an extrusion of the bowel at that part more than elsewhere." He concludes that the chord and hernial sac, when this is not of the congenital variety, are quite separate, or, if in contact, independent of each other. It may be observed that M. Broca,[2] in 1888, investigated the coverings of the ordinary sac and of the sac of the tunica vaginalis, and found that in the first case there is a layer of fascia transversalis between the fibrous sheath of the chord and the sac, whereas in the second case the hernia in the tunica vaginalis is within the coverings of the chord. Dr. Packard appears to confirm M. Broca's observations, but his description is not quite so explicit as that of the French surgeon.

**Sacless Herniæ.**—In the early life of the fœtus the cæcum possesses a complete mesentery, but, after sinking to its final resting-place on the right side of the abdomen, it is sometimes partly denuded of peritoneum, like the ascending colon beyond it. In this condition it has been met with in hernial sacs, and there are a few instances of its being found altogether without serous investment. The sigmoid flexure, likewise, may be partly bare of peritoneum, and four examples have lately been recorded of this anomaly, in which the bowel, thus destitute, formed a left inguinal hernia. Three of these occurred in the practice of Mr. W. Anderson,[3] and a fourth at the Middlesex Hospital under Mr. Morris.[4] This patient was aged 27, and had a left scrotal hernia, for which a radical operation was performed. The sac, when opened, was found to be empty, but a loop of the sigmoid flexure was seen in relief at the back of the cavity behind the serous lining. The meso-sigmoid, in fact, was imperfect, and the single layer attached to the intestine formed part of the posterior wall of the sac. Mr. Morris dissected the bowel and sac from the connective tissue about them. He next divided the sac wall on each side near the bowel, folded the serous membrane backwards around the bare surface of the sigmoid loop, and fixed it in position with sutures. Thus the bowel received a complete covering of serous membrane, provided in part by natural and in part by artificial means. The sigmoid was reduced, the operation concluded, and the patient recovered.

**Vesical Herniæ.**—Mr. Wright, of Manchester,[5] relates a case of strangulated inguinal hernia, in which, after reduction of the bowel, he opened what seemed to be a second sac. This was ligatured and removed, but after death it proved to be a thin-walled diverticulum of the bladder which had protruded through the same opening as the hernia to the inner side of the epigastric artery. Besides these direct herniæ a sac of an oblique hernia was found in the canal emerging through the internal ring. Death was due to leaking of urine into the peri-vesical space.

A case very similar in character, but with a favourable termination, was under the care of Professor Angerer.[6] The man, æt. 56, was admitted to the Surgical Hospital, Munich, for a strangulated left inguinal hernia. Three weeks after herniotomy he desired to be treated for a right hernia, and a radical cure was undertaken. This hernia had been noticed at the age of 42, had formed gradually, and had never attained large dimensions. It did not increase much on cough, and was about the size of a plum. Whilst separating the coverings of the sac the Professor exposed a lipoma the size of a cherry adjacent to its lower end, and below the lipoma a small cyst filled with clear fluid. There was some difficulty in clearing the sac along the inner border, and during this proceeding there suddenly flowed out a tablespoonful of clear fluid. At first it was supposed that a second cyst had been opened, but further examination revealed a thin muscular layer, not easily recognisable. A finger was passed through the accidental opening, and could be made to touch a sound introduced into the bladder by the urethra. The rent was closed with four silk sutures through the muscular coats, and before the bladder was dropped back the security of the stitches was tested by injecting boracic lotion into the viscus. The hernia was an oblique inguinal, but the bladder had protruded close to the outer edge of the rectus in the position of a direct hernia, and was not covered by peritoneum. A radical cure after Kocher's method was performed on the oblique hernia, and the man made a good recovery. He had never noticed the protrusion to be larger when he held his water or less when he passed his water, nor did pressure on the swelling beget a desire to micturate. He had not had any symptoms pointing to disease of the bladder or urethra. Dr. Carl Maunz, who reports the case, goes on to discuss the different theories that have been put forward as to the cause of these vesical herniæ, but without arriving at a fully satisfactory explanation.

**Radical Cure.**—Professor Kocher[7] has introduced a modification in his operation (Verlagerungs methode) for the radical cure. The method consisted in drawing the isolated sac through an opening made in the aponeurosis of the external oblique muscle above, and external to the site of the inner ring. The sac was then twisted and laid over the anterior wall of the inguinal canal, where it was fixed by the sutures that closed the opening in the abdominal wall. Kocher noticed that among forty-eight cases treated by this method suppuration occurred ten times with destruction of the threads and in part of the sac. To obviate this tendency to sloughing caused by the torsion and flexion of the sac he has abandoned both these procedures. The sac, instead of being twisted, is drawn towards the anterior superior iliac spine and is fixed at its place of exit with three sutures, after which it is cut away. The principal suture is that which closes the deep hernial orifice and at the same time includes the portion of sac that passes through the obliquus externus. Afterwards, as formerly, the inguinal canal is closed by deep sutures.

Professor Krause,[8] at Altona, has practised an operation since 1892, which, though not presenting distinctly new features, possesses the advantage of technical simplicity. A long skin incision is first made, and the sac is exposed and freed from below upwards to the highest point possible. It is then opened in its whole length; one or more fingers are passed into the mouth of the sac, which is drawn well down, whilst an assistant passes a silk ligature across it above the operator's fingers. About 1 c.m. below the silk the sac is cut off, and to prevent the ligature from slipping, the ends are sewn over the stump. After the stump of the sac has been returned the hernial aperture is closed with strong silk sutures, passed in an armed needle, through the muscular and aponeurotic structures of the abdominal wall. The first of these is entered above and external to the inner ring.

In an excellent review of the subject of the radical cure of hernia, Kummer[9] lays stress on the necessity of ligating the sac as high up as possible, and of firmly closing the track of the hernia by sutures through the adjacent muscular and aponeurotic planes. He is not in favour of displacement of the neck of the sac nor of the various pads and cushions that have been formed from the omentum, or from the sac itself, behind the abdominal wall.

**Cure of Large Herniæ.**—Dr. Möller relates the case of a man, æt. 35, under Professor Krause, who had a left inguinal rupture reaching down to the knee. It was as large as two adult heads. After twelve days of treatment this vast hernia was reduced, and a radical cure undertaken. All went well till the tenth day, when high fever set in, and on the thirteenth day he died. The man had chronic nephritis, atheroma of the aorta and coronary arteries and hypostatic pneumonia at the left base.

The danger of operating on large herniæ has long been recognised and has been especially insisted upon in this country by Mr. Mitchell Banks. A valuable essay on this subject has been published by Kramer[10] (Glogan), who has collected as many as 240 examples, including six cases of his own, that have been operated upon since 1879. He divides these herniæ into two groups, viz., those about the size of two fists up to the size of a child's head, and those larger than a child's head. Among the whole number there are twenty-four deaths, whereof seventeen were the consequence of infection of the wound. Kramer considers that the danger increases with the size of the hernia, and that among large herniæ those should be deemed unfit for operation that are complicated by disease of the respiratory, circulatory, or urinary organs, &c. With very large herniæ he omits dissection of the sac as a whole, and confines himself to separating only that part of it near the neck. The omentum is freely resected, and the mouth of the sac as well as the canal is closed with silk or catgut.

Kramer justly remarks that when an operation on a large hernia fails to cure, yet the protrusion is much less difficult to retain with a truss than formerly, an observation which may be extended to relapses after radical cure generally.

**Complications.**—Among the rare complications that are met with during this operation may be noted that which Julliard[11] (Geneva) found in two cases. After reducing the small gut and resecting the omentum, he came upon a large mass of sigmoid flexure, adherent to the sac and irreducible. It was necessary to remove the loop and to reunite the divided ends of the bowel.

**Radical Cure in Children.**—M. Broca[12] records 450 radical cures in infants under fifteen years of age. Of inguinal in boys there were 395, in girls 41, and of umbilical 14. One child died of septic peritonitis. Two hundred and fifty cases were seen after more than six months, and only three had relapsed. M. Broca recommends operation in hospital cases as soon as the child is weaned. M. Phocas[13] gives the same advice, but defers the operation till three years of age in children of the well-to-do.

**Strangulated Hernia.**—The cases of strangulated hernia under Professor Kronlein,[14] at Zurich, between 1881 and 1894, amounting to 276, have been arranged in a table by Dr. Oscar Henggeler, and subjected to a detailed and laborious analysis. Dr. Henggeler first reviews some of the statistics on this subject already before the profession, and then proceeds to give a short history of each case, after which the cases are summarised, and the questions arising therefrom are discussed.

In one of the tables the cases have been classified according to the number of hours during which strangulation lasted. The increase of the mortality according to the duration of strangulation is well exemplified, thus:—

| | | | | |
|---|---|---|---|---|
| After strangulation of | 1 day | 8·09 | per cent. | died. |
| „ „ | 2 days | 22·2 | „ | „ |
| „ „ | 3 „ | 45·5 | „ | „ |
| „ „ | 4 „ | 60 | „ | „ |

It should, however, be observed that about half the cases are brought from the country into Zurich, and thus much valuable time is unavoidably lost before operation. The increase of mortality corresponding to the advance in age is not so strikingly progressive in this table as in the tables of other authors, where larger numbers are dealt with.

The average duration of strangulation in hours is also given for each year 1881 to 1894. If the years are taken in groups, there is a distinct and steady diminution in the duration of strangulation, and if this indicates that the cases are taken in hand with greater promptitude than formerly, the surgical teaching in Switzerland is bearing good fruit.

Since 1892 every herniotomy has been concluded by the performance of Bassini's operation, though now it is so far modified that the chord is not displaced. Some of the earlier cases were done after Czerny's method. In femoral hernia the fascia pectinea is sutured to Poupart's ligament. Of 27 cases of gangrenous or suspected bowel an artificial anus was made in 15, of whom only two recovered, and in 12 resection and immediate suture were done, of whom four recovered. Among the whole number (276) the mortality was 23·2 per cent., which compares favourably with the mortality in the London hospitals. Of the rare cases, the greater number have already been published in C. Brunner's essay in 1889, but the following well deserve mention.

**Anomalous Cases.**—A man, æt. 29, who had not worn a truss, fell from a ladder a distance of six feet, and immediately felt pain and swelling in a left hernia. After six hours' strangulation herniotomy was done, and in the sac was found a loop of small intestines, one metre long, and between the layers of its mesentery a large exudation of clear fluid, in size equal to a child's head. This fluid, which had prevented reduction, was evacuated, and the case terminated successfully. The cæcum was met with in three cases, and the sigmoid flexure in five. A right obturator hernia was diagnosed, and reduced by operation. The sac contained, besides bowel, the right ovary, the fallopian tube, and a small, ill-developed uterus. Death occurred next day, and blood was found in the abdomen, which had proceeded from a mesenteric artery and vein that had been torn through at their junction with the intestine.

The effect of the mode of treating the wound is apparently shown in the following table:—

| | | Cases. | Died. |
|---|---|---|---|
| 1881-1885 ... | Carbolic antisepsis | 57 ... | 38·15 p.c. |
| 1885-1893 ... | Corrosive sublimate antisepsis | 32 ... | 21·1 „ |
| 1893-1895 ... | Asepsis | 59 ... | 16·3 „ |

Henggeler rightly observes that these figures are fallacious, for it is the quality of the cases and not the method of treatment that has the most important influence on the prognosis, a fact which has been emphasised by Gussenbauer. A comparison of this table with a former one, showing the average duration of strangulation, enables Henggeler to make the desired correction. For instance, the maximum mortality (56·2 per cent.) fell on the year 1884, in which also the largest average duration of strangulation is noted (83 hours). The minimum of incarceration was in 1893, and here also is a small mortality.

**Treatment of Gangrene.**—A very ingenious suggestion was made at the French Congress of Surgery in October, 1895, by Guinard[15] for the treatment of gangrenous hernia. It has long been the custom to treat small gangrenous spots by making a tuck in the bowel, and thus placing them within the lumen of the tube. This has suggested to Guinard to invaginate the whole gangrenous loop into the lower end of the bowel. A double row of sutures is then placed in the healthy walls of the gut, which fix the invaginated cylinder in the interior of the intestinal canal. After three or four days the gangrenous part is eliminated, and the *débris* are found in the stools. Guinard has used this method in twelve cases. He sums up: (1) Partial invagination is suitable for all cases in which gangrene does not occupy the whole loop; (2) total invagination is the operation of election whenever a gangrenous loop can be introduced in its whole length into the lower end; (3) the only conditions which render it impracticable are the state of the walls of the bowel (œdema, thickening, rigidity), and the length of the loop.

**Reduction en Masse.**—Dr. J. A. MacDougall[16] includes among surgical cases of interest that of a woman who herself reduced "en masse" a strangulated inguinal hernia. He operated with success for the relief of this condition 14 hours after the accident. The inguinal canal was laid open, and it was seen that there was no displacement at the internal ring, but that the body of the sac had been turned upwards and lodged between the internal oblique and transversalis muscles. Reduction of strangulated hernia by the patient is known to occur in about 34 per cent. of those reduced "en bloc," so that the case derives its singularity not so much from this fact as from the very unusual position of the sac between instead of behind the muscles. Dr. MacDougall goes on to discuss the relative advantages of operating at the site of the hernia and of performing laparotomy, and concludes that "it is safest and best to follow the old rule and the old route."

[1] On the Anat. of Oblique Lng. Her., &c. Trans. Amer. Surg. Asso., 1895. [2] Bull. Soc. Anat., 1888, p. 979. [3] Br. Med. Jour., 1895, ii., p. 967. [4] Lancet, 1895, ii. p. 979. [5] Med. Chron., March, 1895, No. 5, p. 346. [6] Münch. Méd. Wochensc, 1895, No. 32, p. 748. [7] Centralb. fr. Chir., 1895, No. 42, p. 958. [8] Münch. Méd. Wochensc. Dr. A. Möller, 1895, No. 42, p. 978. [9] Rev. Med. de la Suisse Romande, 1895, Sep. 20. [10] Centralb. fr. Chir, 1895, No. 42, p. 959. [11] Gaz. des Hôp., 1895, Oct. 26, p. 1,225. [12] Lyon Méd., 1895, Oct. 27, p. 296. [13] Gaz. des Hôp., 1895, Oct. 26, p. 1,226. [14] Beiträge z Klin. Chir. Tübingen, 1895, 15 Bd., 1 Hft. [15] Gaz des Hôp., 1895, Oct. 26, p. 1,225. [16] Ed. Med. Jour., 1895, Dec., p. 529.

